# High Frequency Hearing Loss and Hyperactivity in DUX4 Transgenic Mice

**DOI:** 10.1371/journal.pone.0151467

**Published:** 2016-03-15

**Authors:** Abhijit Dandapat, Benjamin J. Perrin, Christine Cabelka, Maria Razzoli, James M. Ervasti, Alessandro Bartolomucci, Dawn A. Lowe, Michael Kyba

**Affiliations:** 1 Lillehei Heart Institute and Department of Pediatrics, University of Minnesota, Minneapolis, 55455, United States of America; 2 Department of Biochemistry, University of Minnesota, Minneapolis, 55455, United States of America; 3 Program in Physical Medicine and Rehabilitation, University of Minnesota, Minneapolis, 55455, United States of America; 4 Department of Integrative Biology and Physiology, University of Minnesota, Minneapolis, 55455, United States of America; INSERM UMR S_910, FRANCE

## Abstract

Facioscapulohumeral muscular dystrophy (FSHD) is caused by mutations leading to ectopic expression of the transcription factor DUX4, and encompasses both muscle-related and non-muscle phenotypes. Mouse models bearing this gene represent valuable tools to investigate which pathologies are due to DUX4 expression, and how DUX4 leads to these pathologies. The iDUX4(2.7) mouse contains an X-linked doxycycline-inducible DUX4 gene that shows low level basal expression in the absence of doxycycline, leading to male lethality, generally in embryo, but always before 8 weeks of age. Here, we describe additional non-muscle phenotypes in this animal model. We find that iDUX4(2.7) female carriers are extremely hyperactive, spending large amounts of time ambulating and much less time resting. Rare 3-week old males, although hypophagic, runted and extremely fragile, are capable of high activity, but show periods of catatonic torpor in which animals appear dead and respiration is virtually absent. We also examine a non-muscle phenotype of interest to FSHD, high frequency hearing loss. We find that young iDUX4(2.7) females are significantly impaired in their ability to hear at frequencies above 8 kHz. These phenotypes make the iDUX4(2.7) mouse an attractive model in which to study non-muscle activities of DUX4.

## Introduction

Facioscapulohumeral muscular dystrophy (FSHD) is a genetic disease caused by ectopic expression of an unusual double homeodomain transcription factor, DUX4. The proximate cause of this ectopic expression is an alteration in the chromatin that normally forms at the DUX4 locus, converting it from heavily methylated DNA bearing histone marks of heterochromatin or gene repression to relatively demethylated DNA bearing histone marks associated with gene expression, leading to transcription of the locus [1–4]. The *DUX4* gene is embedded in a macrosatellite repeat array, referred to as *D4Z4* and which is normally present in 50+ copies [5]. The ultimate cause of the chromatin changes that lead to its expression is a complex interplay between repeat copy number and second site mutations or allelic differences that affect the activity of chromatin regulators: in most cases, the *D4Z4* array is contracted to 10 or fewer copies; fewer copies of *D4Z4* predispose to transcription [6, 7]. In some cases, transcription/FSHD can occur with >10 repeats, in which the disease is referred to as FSHD2, although clinically indistinguishable from FSHD1 [4]. Mutation of the chromatin protein *SMCHD1* promotes expression of the array, and causes a large number of cases of FSHD2 [8], and genetic background, specifically allelic differences of other genes, presumably chromatin regulators, also influences expression, particularly in the “gray area” of 7–10 repeats [9]. In addition to variation in array number, the sequence downstream of the array comes in multiple alleles, some of which encode a functional polyA, and FSHD only occurs on such alleles [10].

The protein encoded by the locus is expressed at very low frequencies in *ex vivo* cultured cells from FSHD-affected individuals [11, 12]. Forced expression studies in cells *in vitro* have shown that low levels of *DUX4* interfere with *MyoD* transcription, impair myogenesis *in vitro*, and sensitize cells to oxidative stress [13], while high levels of expression lead to cell death [13, 14]. DUX4 binds an AT-rich motif [15, 16] and induces many target genes [13, 15, 17] but a clear explanation for how these molecular events drive muscle wasting is lacking. Three DUX4 transgenic mouse models have been described to date. In the first two, human genomic DNA constructs carrying permissive alleles, one from a two-unit FSHD case and another from a 12 unit control, were integrated randomly to generate high copy and a low copy strains. Neither mouse showed any major pathology, however the low copy strain showed low levels of DUX4 expression in some tissues, including in cells from skeletal muscle [18]. In the third model, referred to as iDUX4(2.7), a 2.7 kb fragment of the *DUX4* gene including the *DUX4* ORF and downstream untranslated sequence was integrated into euchromatic DNA on the X-chromosome, under the regulation of a doxycycline-inducible promoter [19]. In the absence of doxycycline, this transgene showed extremely low levels of basal expression, which caused very strong phenotypes, resulting in very low numbers of live-born males, and male-specific lethality. Females were less affected because of X-inactivation, and although somewhat runted and displaying a skin phenotype, the females were able to propagate the strain by breeding with wild type (WT) males. While muscle tissues from affected males showed no obvious signs of dystrophy prior to their early demise (between 2–8 weeks), they were composed of smaller-diameter fibers, and *ex vivo* cultured myoblasts showed a moderate decrease in proliferation rate. When the *DUX4* transgene was switched on by doxycycline treatment, myoblast differentiation was impaired *in vitro* and skeletal muscle regeneration was impaired *in vivo*. One of the interesting potentially FSHD-related non-muscle phenotypes seen in this mouse model was a retinal vascular telangiectasia. Most FSHD-affected individuals suffer a similar subclinical retinal vasculopathy involving vessel tortuositity and occlusions [20, 21].

In the present study, we have investigated additional phenotypes in these iDUX4(2.7) mice. Because we noted that these animals were highly active, and often had reduced adipose tissue, we investigated their activity levels, metabolism and heat production compared to WT mice. In addition, because high frequency hearing loss is reported as one of the common non-muscle manifestations of FSHD [21], particularly in cases with very short D4Z4 arrays [22], we investigated hearing loss in these mice.

## Results and Discussion

The doxycycline-inducible *DUX4* transgene was targeted into a site on the X-chromosome, therefore the pathological phenotypes are less severe in females than in males [19]. Phenotypes are not due to insertional mutagenesis, as the insertion site is in intergenic DNA upstream of the HPRT gene, which is functional as it was used for selection. X-inactivation mitigates the phenotype in females, as seen most clearly in the skin, where alopecia presents in stripes, typical of Lyonization of X-linked coat color genes in heterozygotes [23], rather than throughout the skin as in males. In other tissues, for example muscle, cells that have inactivated the wild-type (WT) X are apparently selected against during development. Cells from adult female muscle showed almost exclusive inactivation of the DUX4-bearing X [19]. This tissue mosaicism and selection for inactivation of the DUX4-bearing X mean that females present a filtered phenotype. Unfortunately, iDUX4(2.7) males are born alive at only one fifth of the expected Mendelian ratio, they are extremely runted, and rarely live for more than six weeks. These caveats mean that certain phenotypes present most strongly in males, while others are impossible or not meaningful to evaluate in males due to their truncated lifespan, and are thus evaluated in females.

### iDUX4(2.7) males alternate between periods of elevated respiration and episodes of catatonic torpor

**Fig 1:**
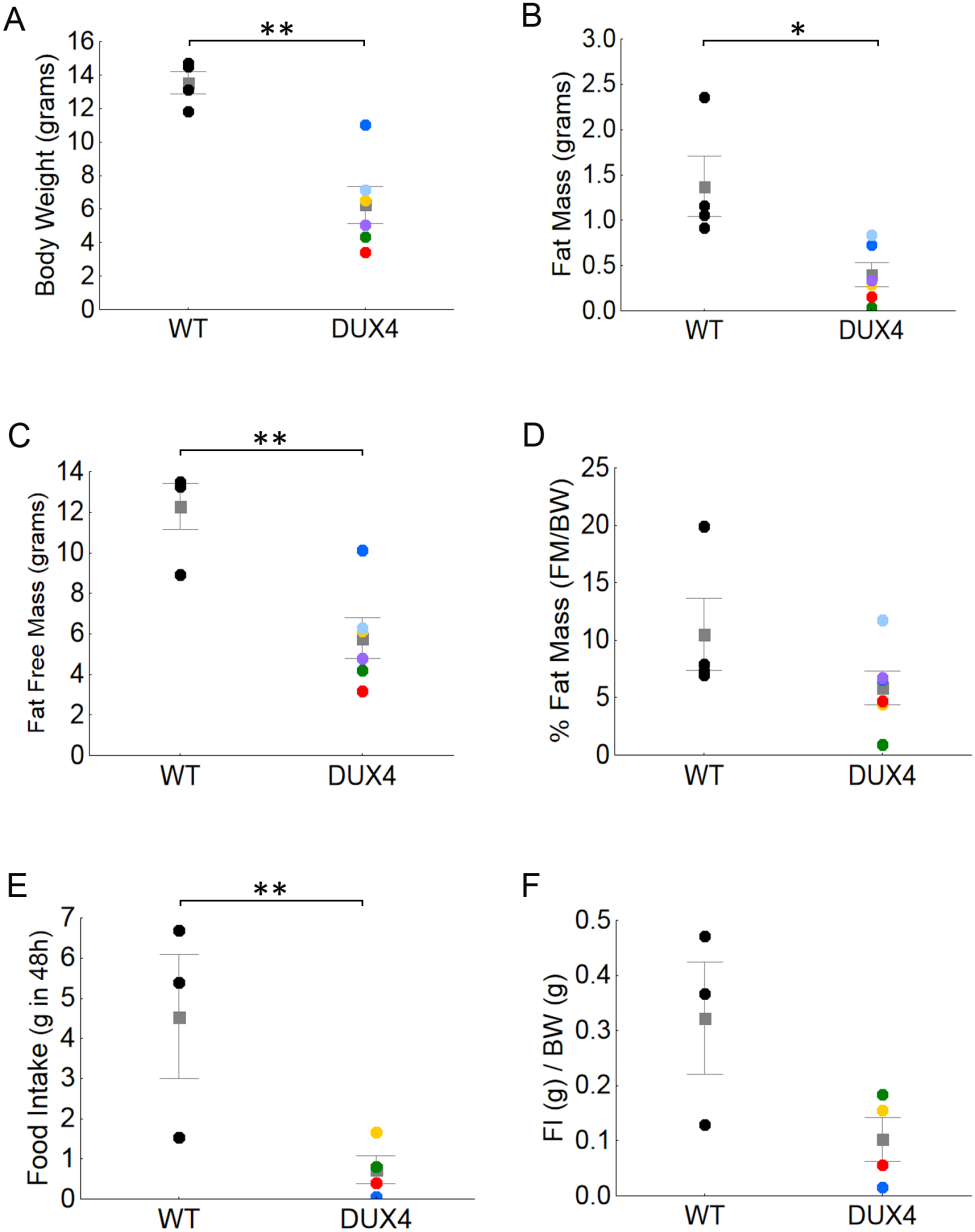
Metabolic parameters in iDUX4 male mice. **A)** Body weight (t_8_ = 4.9, p<0.01). Mean is shown as a gray box; error bars indicate SEM; values for individual mice are indicated by colored symbols. **B**) Fat mass (t_8_ = 3.2, p<0.05). **C**) Fat free mass (t_8_ = 4.2, p<0.01). **D**) % Fat mass. For A-D, n = 4 WT and 6 iDUX4. **E-F**) Food intake during the 48h in the indirect calorimetry chambers expressed in grams (t_5_ = 2.8, p<0.05) and grams relative to body weight. * P<0.05. **P<0.01. Color coding identifies individual DUX4 mice. N = 3 WT and 4 DUX4.

**Fig 2:**
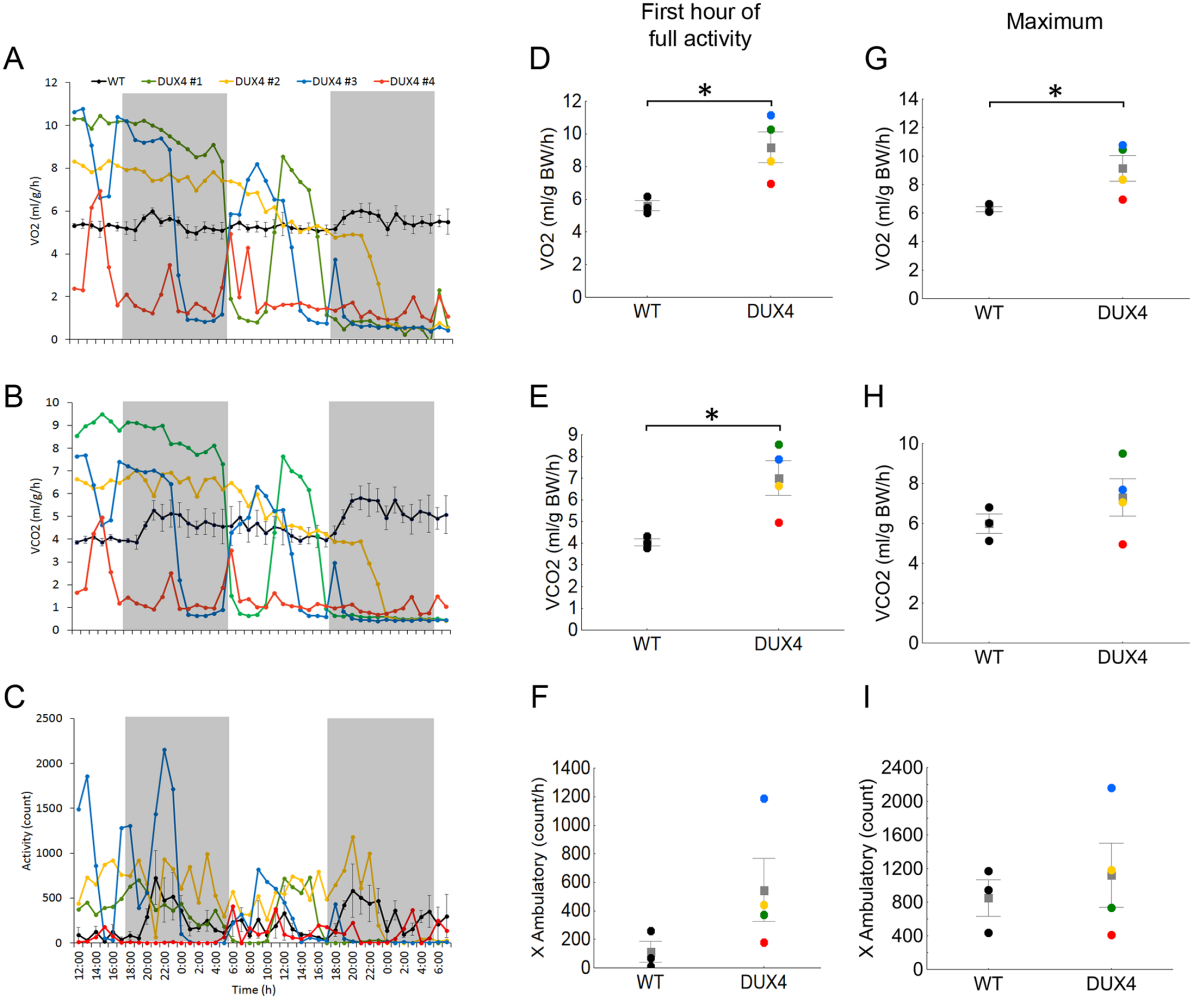
VO_2_, VCO_2_ and locomotor activity in DUX4 male mice at weaning. **A-C)** Hourly average of VO2, VCO2 and locomotor activity measured in the indirect calorimetry chambers over 48h. WT mice are represented by group average, while DUX4 mice are presented individually due to the extreme inter-individual and temporal variability in the metabolic parameters (mice went into and came out of a motionless, energetically negligible state several times over the course of recording). Color coding identifies individual DUX4 animals (colors are consistent with Fig 1). **D-F)** Group averages of metabolic parameters measured during the first hour in which animals showed activity after introducing the animals in the indirect calorimetry chambers. VO2 (t_5_ = 3.1, p<0.05), VCO2 (t_5_ = 3.1, p<0.05). **G-I)** Maximum values recorded during the 48h of recording. (VO_2_ max: t_5_ = 2.7, p<0.05); * P<0.05. For A-I, n = 3 WT and 4 iDUX4.

At weaning, iDUX4(2.7) male mice showed low body size and a high level of variation in body fat (Fig 1). This compelled us to study their metabolism using indirect calorimetry on mice housed for 48 hours in environmental chambers at weaning age. Over these 48 hours, the iDUX4 male weanlings showed a peculiar metabolic phenotype comprised of phases of high VO_2_, VCO_2_, and activity values, interspersed with periods of sudden inactivity coupled with precipitous decline in respiratory exchanges (Fig 2). Most remarkably, after these catatonic torpor-like episodes (prolonged immobility, minimal VO_2_ and VCO_2_) each iDUX4(2.7) animal showed a return to active metabolic function even if they eventually died before the end of the 2 days of recordings (Fig 2). In spite of these phases of prolonged immobility, when the iDUX4(2.7) males were active, they often showed peaks of much greater than normal activity (Fig 2C). Interestingly, during the 2 days in the calorimetry chambers, the iDUX4(2.7) mice also showed lower food intake compared to WT (Fig 1E and 1F), which may implicate food intake as a primary driver of the runting phenotype.

For statistical analysis of metabolic parameters, because many iDUX4(2.7) mice died before the end of the 48 h observation, we compared the values over a one hour period of recording after introducing the mice in the metabolic chambers, and in which no animals were experiencing a catatonic torpor-like episode (these studies exclude one mouse that died before starting the indirect calorimetry). We also evaluated the maximum recorded values for VO_2_, VCO_2_ and activity over the course of the entire 48 h observation. iDUX4(2.7) subjects showed increased maximal VO_2_ compared to WT (Fig 2G) as well as a slight, albeit not significant, increase for all other parameters (Fig 2). Visual inspections of the brown adipose tissue (BAT) showed a small but proportionally normal organ considering the small body size of the DUX4 mice.

We considered the possibility that these metabolic abnormalities were due to deregulated or hyperactive adaptive thermogenesis. We therefore tested whether rearing animals at thermoneutrality (30°C, which is mouse thermoneutrality [24]) would ameliorate the lethal phenotype in males. We acclimatized breeding pairs at thermoneutrality and evaluated frequency and health of live-born male progeny inheriting the *DUX4* transgene. Contrary to the hypothesis, the phenotype was not improved at thermoneutrality. Male pups were still born at much lower than Mendelian ratios, and most died before 3 weeks (Tables 1 and 2). No male carriers survived beyond 6 weeks.

**Table 1:** Viability of male mice at room temperature.

	Live Born Males		Males Surviving to 3 Weeks	
Litters	WT Males	Dux4 Males	WT Survived	Dux4 Survived
#1	4	2	4	
#2	5	1	5	1
#3	4	1	4	
#4	4		4	
#5	4	3	4	
#6	4	1	4	
#7	3		3	
#8	2	1	2	1
**Total**	30	9	30	2
**Ratio**		30%	100%	22%

Summary data from eight litters born at room temperature. Live-born ratios indicate frequency relative to expected (WT males). 3 week survival ratios indicate the frequency of live-born animals surviving to 3 weeks of each genotype.

**Table 2:** Viability of male mice at thermoneutrality.

	Live Born Males		Males Surviving to 3 Weeks	
Litters	WT Males	Dux4 Males	WT Survived	Dux4 Survived
#1	4	1	4	
#2	3	2	3	
#3	3	1	3	1
#4	4		4	
#5	2	1	2	
#6	4	1	4	1
#7	2	1	2	1
#8	3		3	
**Total**	25	7	25	3
**Ratio**		28%	100%	43%

Summary data, as in Table 1, from eight litters born at thermoneutrality.

Due to the low body fat and food intake it is conceivable that the iDUX4(2.7) males die because of insufficient stored nutrients. The metabolic phenotype of these male animals resembles conditions of prolonged fasting [25, 26], H_2_S exposure [27] as well as other mutant strains characterized by small body size and impaired feeding behavior [28, 29]. A type of torpor can be induced by starvation, however such torpor is characterized by periods of inactivity and reduced respiration, not a virtual absence of respiration as was observed in this study. Indeed, based on metabolic parameters, the catatonic mice in this study were presumed to have died, and their recovery considered remarkable when first observed.

### DUX4(2.7) females are hyperactive

**Fig 3:**
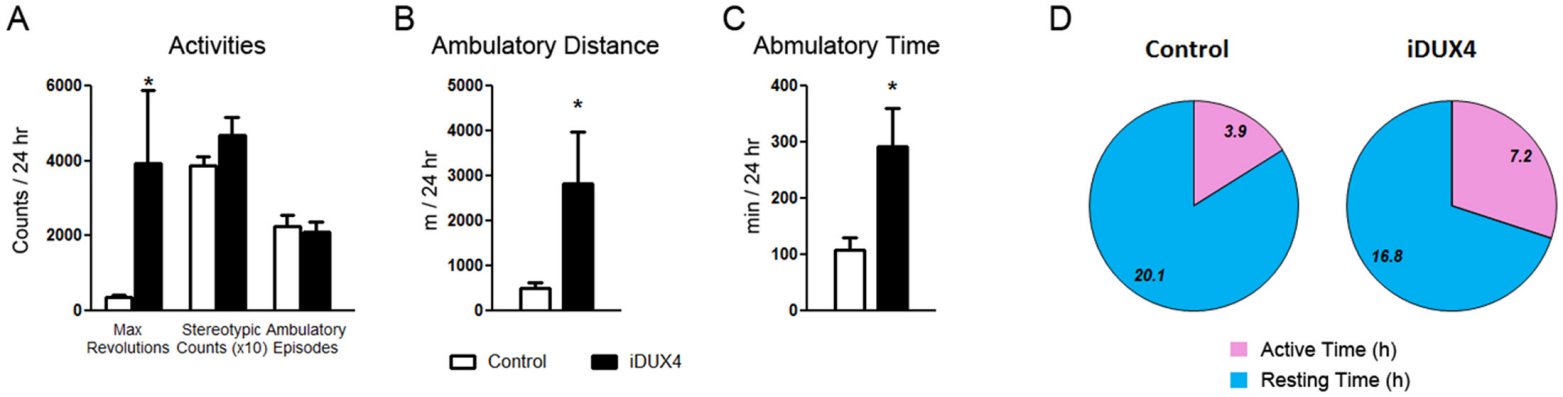
Activity measurements of DUX4 female mice. **A)** Measurements for various types of activity identifiable in activity monitoring cages. For these studies, N = 6 iDUX4(2.7) females and 6 littermate controls. **B)** Total ambulatory distance over 24 hours. **C)** Total ambulatory time over 24 hours. **D)** Comparison of resting *vs*. active time. Differences between control and iDUX4 animals were significant to p = 0.014.

The iDux4(2.7) female mice show a slightly reduced size, but are not severely runted, and they remain relatively healthy and fertile. However, in the course of routine animal husbandry we observed that they were noticeably more jumpy and were often seen spinning. We therefore performed a detailed study of their movements in which 4 month old females and their littermate female controls were analyzed over 24 hours using activity-monitoring cages. This revealed that the female iDUX4(2.7) mice are indeed extremely hyperactive, including abnormal behavioral activities like spinning (Fig 3A). Although the number of ambulatory episodes was not different, the iDUX4(2.7) mice spent much more time engaged in each ambulation and covered more distance (Fig 3A–3C). In addition, comparing active time to time spent resting revealed that the iDUX4 animals spent on average over 7 hours of each day being active compared to < 4h for historical [30] as well as littermate controls (Fig 3D, p = 0.014).

Hyperactivity has not been described in humans with FSHD. We speculated previously [19] that the iDUX4(2.7) mice might represent the extreme high end of a spectrum of phenotypes that arise due to elevated levels of DUX4 expression, because this allele is not subject to repeat-induced silencing (being only a single D4Z4 unit) and because it is inserted into relatively active euchromatin, rather than subtelomeric heterochromatin. Thus, metabolic abnormalities and hyperactivity may require higher levels of DUX4 than are ever seen in FSHD in humans, or they may be novel mouse-specific effects of DUX4.

### DUX4(2.7) females show high frequency hearing loss

**Fig 4:**
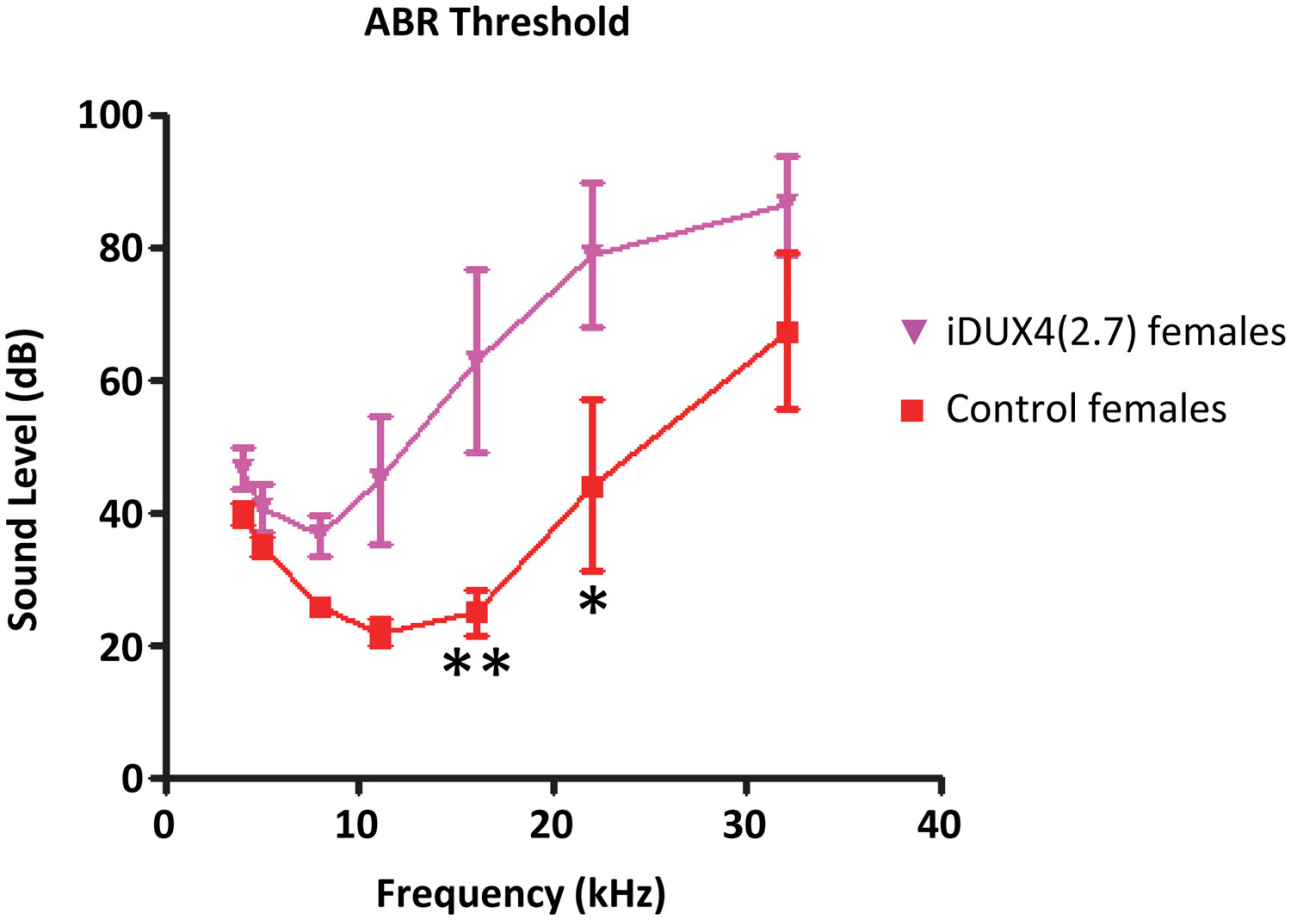
Hearing loss in iDUX4 mice. Auditory brainstem response (ABR) is shown at each frequency indicated over a range of sound levels. N = 5 iDUX4(2.7) and 5 WT littermate control female mice. * p<0.05, **p<0.01.

Because one of the principal non-muscle phenotypes associated with FSHD in humans is high frequency hearing loss [21, 31, 32], we sought to evaluate whether iDUX4(2.7) animals had any hearing abnormalities. Young (between 1 and 5 months of age) female transgenic animals and their WT sibling controls were exposed to 1 msec sonic bursts of increasing amplitude over a range of frequencies and their auditory brainstem response was monitored (Fig 4). This revealed a clear reduction in sensitivity to sound at frequencies greater than 8 kHz. These data are quite interesting from the perspective of non-muscle phenotypes in FSHD, and they suggest that misespression of DUX4 in cells outside of muscle may be responsible for certain pathologies seen in FSHD. Whether this phenotype is homologous to the hearing loss in FSHD patients is difficult to say. Ultimately, the hearing loss in humans and mice are both caused by the *DUX4* gene, but elucidating the mechanism of hearing loss in the iDUX4(2.7) mice will require further study.

## Conclusions

In this study, we document additional dox-independent (i.e. seen in the absence of Dox) phenotypes of the iDUX4(2.7) mice. These include a phenotype of clear relevance to FSHD (hearing loss), as well as phenotypes for which there is no known cognate in this patient population (hyperactivity and periods of apparent complete metabolic inactivity accompanied by immobility).

## Materials and Methods

Mice were maintained in accordance with the Guide for Care and Use of Laboratory Animals of the NIH and the University of Minnesota Institutional Animal Care and Use Committee approved this study. Female iDUX4(2.7) carriers were bred with WT males or with males carrying the Rosa26-rtTA2Sm2 transgene [33].

### Auditory Brainstem Response (ABR)

ABR were performed as described previously [34]. Prior to testing, mice were anaesthetized with avertin and scalp potentials were recorded via subdermal electrodes on the head. Stimuli consisted of symmetrically shaped tone bursts 1 millisecond in duration with 300-microsecond raised cosine ramps and were delivered to a calibrated magnetic speaker. ABRs were bandpass filtered between 0.03 and 10 kHz; signals were amplified 20,000 times, digitized using a 20,000-kHz sampling rate, and subjected to artifact rejection. ABR waveforms were collected for frequencies between 4 kHz and 32 kHz at half octave steps, starting at suprathreshold levels and decreasing in 5 dB steps to a level 10 dB below the threshold. Stacked waveforms were compared and the lowest level of stimulation that evoked an unambiguous ABR waveform was designated as the threshold.

### Activity monitoring cages

Cage activities were measured over 24-h using open field activity chambers (Med Associates Inc., St. Albans, VT) as in Greising et al [30] except for the following. To account for the small size of Dux4 mice, the height of the top horizontal beam was lowered to 2.5 cm and the box size which differentiates stereotypic movement from ambulation was set to “2” (3.2 cm2).

### Metabolic phenotype

Body composition was measured with Echo-MRI (Echo Medical Systems). After body composition measure mice were individually placed in the Oxymax Comprehensive Lab Animal Monitoring System (Columbus Instruments). Oxygen consumption (VO2), carbon dioxide production (VCO_2_) and ambulatory activity were measured over the course of 2 days. Respiratory exchange ratio (RER, or Respiratory quotient) and heat production were calculated but not reported because the assumptions are not met in abnormal metabolic states [35–37]. VO2 and VCO2 were normalized over grams of body weight. Food intake was calculated from the difference between the food allocated to every mouse at the start of the recording and the amount of food that was left at the end of the recording. All experiments were performed at the IBP Phenotyping Core Facility, University of Minnesota. Data were analyzed with unpaired t-test.

### Assessing survival ratios

Litters born at room temperature and 30°C were evaluated daily. Normal mice were weaned at 3 weeks, while runts were kept with their mothers indefinitely. Humane endpoints were used: mice were euthanized by CO2 asphyxiation if they were found to be moribund, i.e. not moving at two different evaluations several hours apart.

### Statistics

Data sets for all experimental and control groups were evaluated for normality (Kolmogorov-Smirnov test) and in where found consistent with normal distribution (most cases), Student’s T-test was used to determine significance of differences between group means, with an assumption of difference at p < 0.05. Mann-Whitney U Tests were used to analyze data that were not distributed normally (cage activity parameters: max revolutions and ambulatory distance) with significance also set at p < 0.05.
